# 8q24 Cancer Risk Allele Associated with Major Metastatic Risk in Inflammatory Breast Cancer

**DOI:** 10.1371/journal.pone.0037943

**Published:** 2012-05-29

**Authors:** François Bertucci, Arnaud Lagarde, Anthony Ferrari, Pascal Finetti, Emmanuelle Charafe-Jauffret, Steven Van Laere, José Adelaide, Patrice Viens, Gilles Thomas, Daniel Birnbaum, Sylviane Olschwang

**Affiliations:** 1 Centre de Recherche en Cancérologie de Marseille, Department of Molecular Oncology, Institut Paoli-Calmettes, Marseille, France; 2 UMR1068 Inserm, Marseille, France; 3 Aix-Marseille Univ, Marseille, France; 4 Fondation Synergie Lyon Cancer, Centre Léon Bérard, Lyon, France; 5 Translational Cancer Research Group, University Hospital, Antwerp, Belgium; University of Edinburgh, United Kingdom

## Abstract

**Background:**

Association studies have identified low penetrance alleles that participate to the risk of cancer development. The 8q24 chromosomal region contains several such loci involved in various cancers that have been recently studied for their propensity to influence the clinical outcome of prostate cancer. We investigated here two 8q24 breast and colon cancer risk alleles in the close vicinity of the *MYC* gene for their role in the occurrence of distant metastases.

**Methodology/Principal findings:**

A retrospective series of 449 patients affected with breast or colon adenocarcinoma was genotyped for the rs13281615 and/or rs6983267 SNPs. Statistical analyses were done using the survival package v2.30 in the R software v2.9.1. The two SNPs did not influence the development of distant metastases of colon cancer; rs6983267 showed a mild effect on breast cancer. However, this effect was greatly emphasized when considering inflammatory breast cancer (IBC) solely. Replicated on a larger and independent series of IBC the contribution of the genotype to the metastatic risk of IBC was found an independent predictor of outcome (p = 2e-4; OR 8.3, CI95∶2.6–33).

**Conclusions/Significance:**

Our study shows first that the monitoring of this specific germline variation may add a substantial tool for IBC prognostication, an aggressive disease that evolves towards distant metastases much more frequently than non-IBC and for which no reliable prognostic factor is available in medical practice. Second, it more generally suggests that risk alleles, while associated with low susceptibility, could correlate with a high risk of metastasis.

## Introduction

The number of treatment options available to patients with breast or colorectal cancer has greatly increased due to a better understanding of cancer biology. Multigene assays have added to the ability to predict disease outcome and degree of response to adjuvant chemotherapy. However a significant proportion of patients with early-stage cancer will develop unpredicted metastatic disease. Candidate genes approaches have identified functional polymorphisms in MMP, PAI-1, HIF-1-alpha, caspases or ACE that moderately influence the risk of invasiveness or metastasis of several cancers, such as those of colon, prostate or lung. More recently, the contribution of cancer susceptibility alleles to clinical outcome has also been evaluated. In prostate cancer patients, an increased frequency of metastasis has been found associated with SNPs genotypes that also increase the risk for cancer itself [1]. These observations indicate that the host genetic constitution may not only contribute to the initial risk of primary tumor development but also to the subsequent risk of metastasis.

Association studies have identified multiple cancer susceptibility loci in a one-megabase region upstream of the *MYC* promoter. Risk alleles have been found for some of the most frequent human carcinomas, prostate, colon and breast, but also for other types such as ovary or bladder [2]–[3]. Several studies have explored the implication of *MYC* in the molecular mechanism underlying this susceptibility. It is a well known target of the WNT signaling pathway that is activated in multiple cancer types, including colon, breast and prostate carcinomas [4]–[6]. The transcription factor TCF7L2/TCF4 has been reported to bind to the rs6983267 region in an allele-specific manner, suggesting that this risk locus may act as part of a *cis*-regulatory enhancer element for *MYC* [7]–[8]. Close to rs6983267 that has been reported to modulate colorectal cancer risk, an independent SNP rs13281615 has been linked to breast cancer risk.

We report here the allelic frequencies of both rs13281615 and rs6983267 in patients affected with colorectal or breast cancer and their behavior according to the disease evolution and the morphologic features of these two cancer types.

## Results

### Genotypes and Metastatic Risk of Colon and Breast Cancers

**Figure 1:**
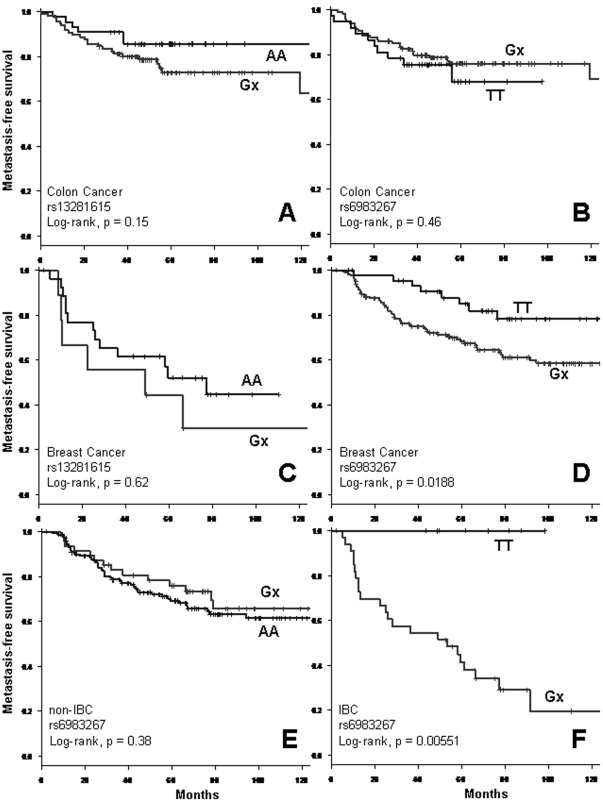
Kaplan-Meier analysis of metastasis-free survival in colon cancers and breast cancers for rs13281615 and rs6983267 genotypes. Comparison of MFS curves for patients with at least one risk allele (Gx) and no risk alleles (AA for rs13281615; TT for rs6983267): in colon cancers at rs13281615 (part A) and at rs6983267 (part B) SNPs; in breast cancers at rs13281615 (part C) and at rs6983267 (part D) SNPs; in non-IBCs (part E), and in IBCs (part F) at rs6983267.

The two 8q24 rs13281615 and rs6983267 SNPs were genotyped in the learning series of 152 colon cancers (Table S1) and 207 breast cancers (Table S2). The frequencies of different alleles were similar to those described in the litterature (Table 1). To analyze the contribution of the risk allele (G allele) to the metastatic process, patients with at least one G allele were compared to those homozygous for the non-risk allele. Search for correlations between the two categories of genotypes at both loci (Gx *vs*. AA or TT) and the 5-year metastasis-free survival (MFS) failed to reveal difference in outcome among the two groups of colon cancers patients (Figure 1A-B). For breast cancers, MFS was not associated with genotype at the rs13281615 locus (Figure 1C). However, the risk allele of the rs6983267 locus was weakly associated with an increase in metastatic risk. At 5 years, the metastatic-free survival was 85% (95% CI 74–97) for patients with the TT genotype *vs.* 68% (95% CI 61–76) for those with the Gx genotype (p<0.02, log-rank test, Figure 1D). Separate analysis of IBC and non-IBC patients did not reveal any difference in survival for non-IBC patients (5-year MFS of 82% *vs*. 76%, p = 0.38, log-rank test, Figure 1E). However, when the analysis was restricted to IBC patients a striking difference in MFS was observed: the TT genotype was associated with a 5-year MFS of 100% (95% CI 100–100) whereas the Gx genotype was associated with a 5-year MFS of 41% (95% CI 27–63) (p = 0.005, log-rank test; Figure 1F).

**Table 1:** Genotyping data at loci rs13281615 and rs6983267 in the learning and the merged IBC series.

SNP	Genotype	Colon cancers^1^	Breast cancers^1^	Inflammatory breast cancers^2^
	GG	47 (31%)	55 (26%)	38 (29%)
rs6983267	GT	68 (45%)	107 (52%)	69 (52%)
	TT	37 (24%)	45 (22%)	25 (19%)
rs13281615	AA	43 (31%)	48 (25%)	10 (25%)
	AG	72 (52%)	104 (54%)	22 (55%)
	GG	24 (17%)	39 (21%)	8 (20%)

1 learning sets;

2 merged learning and validation sets.

**Figure 2:**
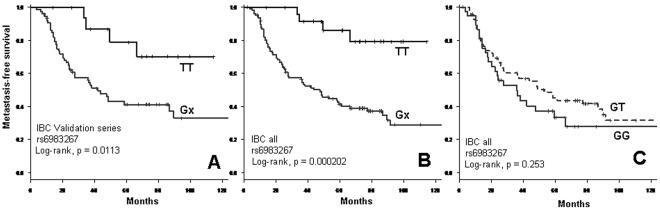
Kaplan-Meier analysis of metastasis-free survival in IBCs for rs6983267. Parts A and B. Comparison of MFS curves for IBC patients with at least one risk allele (Gx) and no risk alleles (TT) at rs6983267 in the validation set (part A) and in the pooled learning and validation sets (part B). Part C. Comparison of MFS curves for all IBC patients with at least one risk allele (Gx) according to the genotype GG *vs.* GT.

To validate the MFS association observed with the rs6983267 locus in this first series of 42 IBC, an independent series of 90 IBC samples was analyzed using the same experimental approach. As shown in Figure 2A, the 5-year MFS of IBC patients displayed a similar pattern, with 79% (95% CI 60–100) for patients of TT genotype and 41% (95%CI 31–55) for those bearing at least one G allele (p = 0.0113, log-rank test). The two IBC series were then merged into a single one (Table S3). Regarding the rs6983267 SNP, 25 patients were of TT genotype (19%) and 107 of Gx genotype (81%). Correlations of genotypes and histo-clinical factors were assessed (Table S4). Genotype was not associated with any of the histo-clinical factors (age, histological type and grade, estrogen receptor (ER), progesterone receptor (PR) and ERBB2 status, pathological response to chemotherapy) but with MFS: 66 of the 107 Gx patients (62%) displayed metastatic relapse *vs* only 4 of the 25 (16%) TT patients (p = 3.8E-05, Fisher’s exact test). The 5-year MFS was 41% in the case of a Gx genotype *vs*. 86% in the case of a TT genotype (p = 0.0002, log-rank test; Figure 2B). Within the 107 Gx patients, those of GG (38 cases) and those of GT (69 cases) genotype did have a similar behavior (Figure 2C).

### Prognostic Value of Rs6983267 SNP in Inflammatory Breast Cancer

Univariate and multivariate analyses of the MFS were then done to compare the prognostic performance of the rs6983267 genotype with that of histo-clinical factors in IBC (Table S5). In univariate analysis, the hazard ratio (HR) for metastatic relapse was 5.56 when comparing Gx to TT genotypes (95% CI [2.00–14.3], p = 0.0009). The PR status was also associated with MFS, whereas ER and ERBB2 status and the pathological response only tended to be associated with the outcome. Information regarding the three variables showing significance with a p-value lower than 0.1 in univariate analysis (*i.e.* genotype, PR and ER status) was available for 130 of the 132 samples thus making possible a multivariate analysis. In the latter, PR status lost its prognostic significance and ER remained not significant. Only the rs6983267 genotype remained significant.

### Gene Expression and Genome Profiles in IBC According to the Rs6983267 Genotype

We next wanted to determine whether differences in genotype could be associated with specific characteristics. We searched for differences in RNA expression profiles in IBC samples classified according to their rs6983267 genotype (Gx *vs*. TT) or their metachronous metastatic status (presence or absence) using a previously published data set (GEO accession number GSE23720); no significant difference was found; in particular, a difference in *MYC* mRNA expression was not detected. Similarly, array-based comparative genomic hybridization failed to detect specific genomic profiles [9]–[10]; again, no difference in amplification level of the 8q24 region around *MYC* was observed and thus, no candidate gene emerged at this stage. However it had previously been shown that the control of *MYC* expression was subtle when studying the possible influence of the genotype on *MYC* expression level [7], [8], [11]–[13].

## Discussion

This association study globally showed that the two independent SNPs, rs13281615 and rs6983267, which modulate the risk of breast and colorectal cancer respectively, have no major influence on the development of distant metastases in colon cancer. However, it unexpectedly revealed that the rs6983267 genotype strongly influences the metastatic risk of an aggressive form of breast cancer, namely inflammatory breast cancer. Because the metastatic risk was found comparable for patients of GT and GG genotypes, the G allele is likely to play a dominant role over the T allele. The absence of the G allele reduced the metastatic risk to a level similar to that of non-IBCs patients. The presence of the G allele in a context of inflammatory tumor might thus potentiate a specific metastatic mechanism that would superimpose to the « standard » metastatic risk of breast cancer. With an odd ratio of 8.3 (CI95: 2.6–33), the prognostic value of the G allele might add to the predictive value derived from the currently limited pre-therapeutic evaluation of IBC patients. It might be interesting to use this feature in prospective studies. For instance, the monitoring of the rs6983267 genotype of IBC patients would enable to focus the analysis on subgroups of patients with comparable risk for metastasis. Because genotypic studies can be done retrospectively, this approach might not only be applicable to future studies but also to past surveys.

The alleles of rs6983267 are known to differentially bind transcription regulating elements such TCF7L2/TCF4 [12]–[13]. The downstream activation of corresponding snpRNAs have also been involved in the phenotypic orientation of prostate cancer [14]. We found the presence of a large chromatin-loop bringing the rs6983267 in the vicinity of *MYC* in two IBC cell lines SUM149 and SUM190 (unpublished observations done in collaboration with Matthew Freedman) suggesting that the increased metastatic risk of inflammatory tumors might be promoted by a deregulation of the *MYC* gene, as it was initially proposed for colon cancer [4]. Because IBC is defined by the presence of tumor emboli within dermal lymphatic vessels and as it was recently shown that vessels inflammation could be regulated by beta-catenin-independent signaling pathway involving WNT5A and TNF alpha signaling [15], IBC specificity could be linked to WNT/TCF4/MYC pathway activation in inflammatory lymphatic vessels.

In conclusion, the rs6983267(G) allele, initially described as a low penetrance susceptibility locus for colorectal and prostate cancers, appears to be associated with a high risk for metastasis in inflammatory breast cancer. The implementation of new tools for tumor prognostication, presently based on the histo-clinical factors and somatic mutations, may benefit from the monitoring of specific germline variations of the IBC patients. To date, no candidate gene emerges from expression studies, but *MYC* remains a good one as it is able to binds both alleles of rs6983267 through a large chromatin-loop in two IBC cell lines. Further functional studies have thus to delineate this physical link. Although IBC do not exhibit a specific expression profile, they tend to aggregate poor prognosis breast cancer clusters, being commonly of basal-like subtype, showing Her-2 amplification, EGFR overexpression and lacking estrogen and progesterone receptors. Variations in the *NFKB1* and *COX2* genes expression linked to *HER-2* and *EGFR* expression levels might be interesting arguments to propose targeted therapies [16]. Finally, other known cancer risk loci could be associated with metastatic risk in subgroups of cancer and new association studies may identify more susceptibility loci to metastasis.

## Materials and Methods

### Ethic Statement

All patients signed a written informed consent and the study was approved by our institutional review board.

### Biological Material

Tumor tissue samples were collected from 359 patients with invasive adenocarcinomas, who underwent surgical biopsies or initial surgery at the Institut Paoli-Calmettes. Patients were generally native from the Mediterranean basin mainly including North-Africa and Italy. These samples constituted the learning series including breast and colon cancers selected from the Data Management Centre of Institut Paoli-Calmettes (Marseille, France). Selection criteria included the availability of a pre-treatment genomic DNA sample, no distant metastasis at diagnosis and long-term follow-up (Tables S1 and S2).

Additional samples were collected from patients affected with inflammatory breast cancer (IBC) to validate the results observed in the learning set. IBC presented with diffuse erythema, associated with a local sensation of heat and edema, induration and the typical “peau d’orange” aspect of the skin. Skin biopsies that were systematically performed at diagnosis objectived the blockage of lympahtic channels due to tumor emboli [16]. A total of 76 new samples from IBC patients, non-metastatic at diagnosis, were retained from the IBC database of Institut Paoli-Calmettes, because of the availability of fixed tissue sample in the Biopathology department. Because IBC is a rare disease that contributes for less than 5% of breast cancer fourteen non-metastatic cases collected from patients treated at the Antwerp University Hospital (Belgium) were also recruited to enlarge the series, leading to a validation series of 90 IBC cases. None of these 90 samples was included in the learning series. The 132 samples represents one of the largest series of IBC ever constituted. The characteristics of all IBC from both learning and validation series are presented in additional table 3.

### Genotyping

Genomic DNA was extracted from either frozen or formalin-embedded fixed material using the DNA QIAamp® micro-kit as recommended by the manufacturer. The quality and quantity of extracted DNA were evaluated with a NanoDrop ND-1000 spectrophotometer (Thermo Fisher Scientific, Illkirch France). The rs13281615 and rs6983267 loci were double-strand sequenced after two rounds of PCR amplification using the 5′GGACCGTAGCTTCTGTATCTGCAA/5′ AGTGTCTTTATGGCCCTAGCAG and 5′TCCTATCTCAGCTCCCTATCCA/5′ CCCAATCCTGAGAAACTTGC pairs of primers at 58°C and 55°C for annealing, respectively. To validate genotypic data derived from fixed material, IBC samples from the learning series were also characterized on fixed material. No discordance was noticed.

### Statistical Analyses

Correlations between sample groups and histo-clinical factors were calculated with the Fisher’s exact test for qualitative variables with discrete categories. Follow-up was measured from the date of diagnosis to the date of last news for patients without any metastatic relapse. Metastasis-free survival (MFS) was calculated from the date of diagnosis until the date of first distant relapse using the Kaplan-Meier method. Survival was compared between groups with the log-rank test. Univariate and multivariate analyses were done using Cox regression analyses. The p-values were based on the Wald test and patients with one or more missing data were excluded. All statistical tests were two-sided at the 5% level of significance. Analyses were done using the survival package (version 2.30) in the R software (version 2.9.1).

## Supporting Information

Table S1 **Histo-clinical characteristics of the colon cancer series.** 1, rectal cancers were excluded. After colonic surgery, patients were treated according to standard guidelines; 54% received adjuvant 5-fluoro-uracil-based chemotherapy. The median follow-up of patients without any metastatic relapse was 58 months after diagnosis. A total of 37 patients experienced a metastatic relapse. The 5-year metastasis-free survival (MFS) was 74% (95%CI 67–82).
(DOC)

Table S2 **Histo-clinical characteristics of the breast cancer series.** 1, The breast samples were obtained from surgical biopsies or initial surgery. Positive immunohistochemical (IHC) status for ER and PR was defined by 10% or more stained tumor cells; ERBB2-positive status was defined as 3+score using the DAKO HercepTest, or 2+score complemented with Fluorescent *In Situ* Hybridisation (FISH) amplification (HER2/CEP17 ratio higher than 2.2). Due to a specific referral to our institution, the 207 cases comprised 42 inflammatory breast cancers (IBC) defined upon clinical criteria as T4d tumors and 165 non-inflammatory breast cancers (non-IBCs). Patients were treated according to standard guidelines: 99% of patients had surgery and 99% received adjuvant radiotherapy. All patients received adjuvant and/or neo-adjuvant chemotherapy and 50% received adjuvant hormone therapy. The median follow-up of patients with no metastatic relapse was 80 months after diagnosis. A total of 66 patients experienced a metastatic relapse. The 5-year MFS was 72% (95%CI 66–79); 2, IDC, invasive ductal cancer; ILC, invasive lobular cancer; IBC, inflammatory breast cancer; 3, concerns non-IBC only.
(DOC)

Table S3 **Histo-clinic characteristics of the merged IBC series.** 1, All patients were treated with primary chemotherapy and most of them with surgery and radiotherapy. After completion, adjuvant hormone therapy was given to 58% of them. With a median follow-up of 72 months after diagnosis, the 5-year MFS was 50% (95% CI 41–59); 2, IDC, invasive ductal cancer; ILC, invasive lobular cancer.
(DOC)

Table S4 **rs6983267 genotyping and histo-clinical correlations in the merged series of IBC.**
(DOC)

Table S5 **Uni- and multivariate logistic regression analyses for MFS in the merged IBC series.**
(DOC)
