# Supplementary material for: 8q24 Cancer Risk Allele Associated with Major Metastatic Risk in Inflammatory Breast Cancer
Source: PLoS One. 2012 May 29;7(5):e37943. doi: 10.1371/journal.pone.0037943 (PMC3362533; doi:10.1371/journal.pone.0037943)
Supplement: Table S1 — Histo-clinical characteristics of the colon cancer series. 1, rectal cancers were excluded. After colonic surgery, patients were treated according to standard guidelines; 54% received adjuvant 5-fluoro-uracil-based chemotherapy. The median follow-up of patients without any metastatic relapse was 58 months after diagnosis. A total of 37 patients experienced a metastatic relapse. The 5-year metastasis-free survival (MFS) was 74% (95%CI 67–82). (DOC) [file pone.0037943.s001.doc]

**Table S1: Histo-clinical characteristics of the colon cancer series**

| **Characteristics (N)^1^** | **N (%)** |
| --- | --- |
| Age at diagnosis (152) | |
| ≤50 years | 18 (12%) |
| >50 years | 134 (88%) |
| Sex (152) | |
| F | 78 (51%) |
| M | 74 (49%) |
| Location (152) | |
| Right | 58 (38%) |
| Left | 93 (61%) |
| Multiple | 1 (1%) |
| Stage (152) | |
| 2 | 104 (68%) |
| 3 | 48 (32%) |
| Surgery | |
| no | 0 (0%) |
| yes | 152 (100%) |
| Adjuvant chemotherapy (152) | |
| no | 70 (46%) |
| yes | 82 (54%) |
| Follow-up (median) (152) | 57.7 |
| Metastatic relapse (152) | |
| no | 115 (76%) |
| yes | 37 (24%) |
| 5y-MFS (152) | 74% [0.67-0.82] |
