# Supplementary material for: 8q24 Cancer Risk Allele Associated with Major Metastatic Risk in Inflammatory Breast Cancer
Source: PLoS One. 2012 May 29;7(5):e37943. doi: 10.1371/journal.pone.0037943 (PMC3362533; doi:10.1371/journal.pone.0037943)
Supplement: Table S2 — Histo-clinical characteristics of the breast cancer series. 1, The breast samples were obtained from surgical biopsies or initial surgery. Positive immunohistochemical (IHC) status for ER and PR was defined by 10% or more stained tumor cells; ERBB2-positive status was defined as 3+score using the DAKO HercepTest, or 2+score complemented with Fluorescent In Situ Hybridisation (FISH) amplification (HER2/CEP17 ratio higher than 2.2). Due to a specific referral to our institution, the 207 cases comprised 42 inflammatory breast cancers (IBC) defined upon clinical criteria as T4d tumors and 165 non-inflammatory breast cancers (non-IBCs). Patients were treated according to standard guidelines: 99% of patients had surgery and 99% received adjuvant radiotherapy. All patients received adjuvant and/or neo-adjuvant chemotherapy and 50% received adjuvant hormone therapy. The median follow-up of patients with no metastatic relapse was 80 months after diagnosis. A total of 66 patients experienced a metastatic relapse. The 5-year MFS was 72% (95%CI 66–79); 2, IDC, invasive ductal cancer; ILC, invasive lobular cancer; IBC, inflammatory breast cancer; 3, concerns non-IBC only. (DOC) [file pone.0037943.s002.doc]

**Table** S2: Histo-clinical characteristics of the breast cancer series

| Characteristics (N)^1^ | N (%) |
| --- | --- |
| Age (207) | |
| ≤50 years | 113 (55%) |
| >50 years | 94 (45%) |
| Histological type (201)^2^ | |
| IDC | 159 (79%) |
| ILC | 11 (5%) |
| Other | 31 (15%) |
| Clinical form | |
| IBC | 42 (20%) |
| non-IBC | 165 (80%) |
| SBR grading (204) | |
| 1 | 21 (10%) |
| 2 | 64 (31%) |
| 3 | 119 (58%) |
| Pathological tumour size^3^ (pT) (159) | |
| pT1 | 30 (19%) |
| pT2 | 88 (55%) |
| pT3 | 41 (26%) |
| Pathological axillary lymph node status^3^ (pN) (161) | |
| neg | 58 (36%) |
| pos | 103 (64%) |
| IHC ER status (207) | |
| neg | 93 (45%) |
| pos | 114 (55%) |
| IHC PR status (207) | |
| neg | 108 (52%) |
| pos | 99 (48%) |
| ERBB2 status (189) | |
| neg | 147 (78%) |
| pos | 42 (22%) |
| Surgery (196) | |
| no | 1 (1%) |
| yes | 195 (99%) |
| Radiotherapy (207) | |
| no | 3 (1%) |
| yes | 204 (99%) |
| Chemotherapy (207) | |
| no | 0 (0%) |
| yes | 207 (100%) |
| Hormone therapy (207) | |
| no | 104 (50%) |
| yes | 103 (50%) |
| Median follow-up (months) (207) | 80.2 |
| Metastatic relapse (207) | |
| no | 141 (68%) |
| yes | 66 (32%) |
| 5y-MFS (207) | 72% [66-79] |
