# Supplementary material for: 8q24 Cancer Risk Allele Associated with Major Metastatic Risk in Inflammatory Breast Cancer
Source: PLoS One. 2012 May 29;7(5):e37943. doi: 10.1371/journal.pone.0037943 (PMC3362533; doi:10.1371/journal.pone.0037943)
Supplement: Table S3 — Histo-clinic characteristics of the merged IBC series. 1, All patients were treated with primary chemotherapy and most of them with surgery and radiotherapy. After completion, adjuvant hormone therapy was given to 58% of them. With a median follow-up of 72 months after diagnosis, the 5-year MFS was 50% (95% CI 41–59); 2, IDC, invasive ductal cancer; ILC, invasive lobular cancer. (DOC) [file pone.0037943.s003.doc]

**Table S3: Histo-clinic characteristics of the** merged IBC series

| **Characteristics (N)^1^** | **N (%)** |
| --- | --- |
| Age (132) |  |
| ≤50 years | 77 (58%) |
| >50 years | 55 (42%) |
| Histological type^2^ (132) | |
| IDC | 118 (89%) |
| ILC | 7 (5.5%) |
| Other | 7 (5.5%) |
| SBR grading (129) | |
| 1 | 5 (4%) |
| 2 | 38 (29%) |
| 3 | 86 (67%) |
| IHC ER status (130) | |
| neg | 56 (43%) |
| pos | 74 (57%) |
| IHC PR status (130) | |
| neg | 66 (51%) |
| pos | 64 (49%) |
| ERBB2 status (121) | |
| neg | 75 (62%) |
| pos | 46 (38%) |
| Pathological complete response (pCR) (76) | |
| no | 49 (64%) |
| yes | 27 (36%) |
| rs6983267 (132) | |
| GG | 38 (29%) |
| GT | 69 (52%) |
| TT | 25 (19%) |
| rs6983267 (132) | |
| Gx | 107 (81%) |
| TT | 25 (19%) |
| Chemotherapy (132) | |
| no | 0 (0%) |
| yes | 132 (100%) |
| Surgery (132) | |
| no | 5 (4%) |
| yes | 127 (96%) |
| Radiotherapy (132) | |
| no | 3 (2%) |
| yes | 129 (98%) |
| Hormone therapy (132) | |
| no | 56 (42%) |
| yes | 76 (58%) |
| Follow-up (median) (132) | 72,5 |
| Metastatic relapse (132) | |
| no | 62 (47%) |
| yes | 70 (53%) |
| 5y-MFS (132) | 50 [41.0-59.0] |
