# Supplementary material for: 8q24 Cancer Risk Allele Associated with Major Metastatic Risk in Inflammatory Breast Cancer
Source: PLoS One. 2012 May 29;7(5):e37943. doi: 10.1371/journal.pone.0037943 (PMC3362533; doi:10.1371/journal.pone.0037943)
Supplement: Table S4 — rs6983267 genotyping and histo-clinical correlations in the merged series of IBC. (DOC) [file pone.0037943.s004.doc]

**Table S4**: rs6983267 genotyping and histo-clinical correlations in the whole series of IBC

| Characteristics (N) | Gx genotype (N=107) | TT genotype (N=25) | *p* | OR |
| --- | --- | --- | --- | --- |
| Age (132) | | | 0.37 | 0.6 (0.21-1.63) |
| ≤50 years | 60 (56%) | 17 (68%) |  |  |
| >50 years | 47 (44%) | 8 (32%) |  |  |
| Histological type (132) | | | 0.33 |  |
| IDC | 95 (89%) | 23 (92%) |  |  |
| ILC | 7 (7%) | 0 (0%) |  |  |
| Other | 5 (5%) | 2 (8%) |  |  |
| SBR grading (129) | | | 0.71 |  |
| 1 | 5 (5%) | 0 (0%) |  |  |
| 2 | 31 (30%) | 7 (28%) |  |  |
| 3 | 68 (65%) | 18 (72%) |  |  |
| IHC ER status (130) | | | 0.067 | 2.66 (0.92-8.85) |
| neg | 50 (47%) | 6 (25%) |  |  |
| pos | 56 (53%) | 18 (75%) |  |  |
| IHC PR status (130) | | | 0.072 | 2.4 (0.88-7.07) |
| neg | 58 (55%) | 8 (33%) |  |  |
| pos | 48 (45%) | 16 (67%) |  |  |
| ERBB2 status (121) | | | 0.24 | 0.51 (0.15-1.52) |
| neg | 58 (59%) | 17 (74%) |  |  |
| pos | 40 (41%) | 6 (26%) |  |  |
| Pathological complete response (pCR) (76) | | | 0.56 | 1.55 (0.42-5.48) |
| no | 40 (67%) | 9 (56%) |  |  |
| yes | 20 (33%) | 7 (44%) |  |  |
| Metastatic relapse (132) | | | **3.83E-05** | 0.12 (0.03-0.39) |
| no | 41 (38%) | 21 (84%) |  |  |
| yes | 66 (62%) | 4 (16%) |  |  |
|  |  |  |  |  |
| 5y-MFS (132) | 41% [33-52] | 86% [72-100] | **2.02E-04** |  |
| Follow-up (median) (132) | 73.8 | 72.3 | 0.35 |  |
