# Supplementary material for: 8q24 Cancer Risk Allele Associated with Major Metastatic Risk in Inflammatory Breast Cancer
Source: PLoS One. 2012 May 29;7(5):e37943. doi: 10.1371/journal.pone.0037943 (PMC3362533; doi:10.1371/journal.pone.0037943)
Supplement: Table S5 — Uni- and multivariate logistic regression analyses for MFS in the merged IBC series. (DOC) [file pone.0037943.s005.doc]

**Table S5**: Uni- and multivariate logistic regression analyses for MFS in the merged IBC series

| Parameter | Comparison | Univariate | | | Multivariate | | |  |
| --- | --- | --- | --- | --- | --- | --- | --- | --- |
|  |  | N | HR [95CI] | p | N | HR [95CI] | p | |
| Age (years) | >50 *vs.* ≤50 | 132 | 1.27 [0.75-2.03] | 0,32 |  |  |  | |
| Histological type |  | 132 |  | 0,69 |  |  |  | |
|  | ILC *vs.*IDC |  | 1.24 [0.45-3.42] |  |  |  |  | |
|  | Other *vs.*IDC |  | 1.44 [0.58-3.59] |  |  |  |  | |
| SBR grading |  | 129 |  | 0,79 |  |  |  | |
|  | 2 *vs.*1 |  | 1.26 [0.38-4.25] |  |  |  |  | |
|  | 3 *vs.*1 |  | 1.06 [0.33-3.42] |  |  |  |  | |
| IHC ER status | pos *vs.*neg | 130 | 0.63 [0.40-1.02] | 0,06 | 130 | 1.00 [0.55-1.82] | 0,99 | |
| IHC PR status | pos *vs.*neg | 130 | 0.53 [0.33-0.85] | 0,0091 | 130 | 0.57 [0.31-1.06] | 0,07 | |
| ERBB2 status | pos *vs.*neg | 121 | 1.45 [0.88-2.40] | 0,14 |  |  |  | |
| pCR | yes *vs.*no | 76 | 0.59 [0.29-1.21] | 0,15 |  |  |  | |
| SNP rs6983267 | Gx *vs.*TT | 132 | 5.56 [2.00-14.3] | 0,00095 | 130 | 5.00 [1.79-14.3] | 0,0021 | |
